# Blink rate during saccadic eye movements: insights from patients with chronic pain

**DOI:** 10.25122/jml-2024-0271

**Published:** 2024-03

**Authors:** Emanuel Ştefănescu, Ştefan Strilciuc, Vlad-Florin Chelaru, Diana Chira, Dafin Mureşanu

**Affiliations:** 1 Department of Neuroscience, Iuliu Hațieganu University of Medicine and Pharmacy, Cluj-Napoca, Romania; 2 RoNeuro Institute for Neurological Research and Diagnostic, Cluj-Napoca, Romania; 3 Research Center for Functional Genomics, Biomedicine, and Translational Medicine, Iuliu Hațieganu University of Medicine and Pharmacy, Cluj-Napoca, Romania

**Keywords:** chronic pain, eye-tracking, blink rate

## Abstract

Chronic pain (CP) significantly impacts quality of life and poses an increasing economic burden on healthcare systems worldwide. This study investigates the relationship between blink rate during saccadic eye movements and pain perception in patients with CP. Ninety-two patients with CP (24 men, 68 women) were assessed using eye-tracking technology during horizontal and vertical saccadic tasks. Pain perception was evaluated using the Central Sensitization Inventory - Part A and the McGill Pain Questionnaire, and anxiety levels were measured using the State-Trait Anxiety Inventory. The results revealed a significant correlation between blink rates in horizontal and vertical tasks (ρ = 0.668, *P* < 0.001). However, there was no correlation between blink rate and age, pain perception, or anxiety scores. Conversely, measures of pain perception were positively correlated with each other and with anxiety scores. These findings suggest that while blink rate may reflect oculomotor function, it is not directly influenced by pain perception or anxiety in patients with CP. The present study highlights the potential of eye tracking for the comprehensive assessment and management of patients with CP.

## INTRODUCTION

Chronic pain (CP) is a significant personal and economic burden, affecting over 30% of the global population. In the United States alone, the estimated direct and indirect costs attributable to CP exceed $600 billion annually. Higher prevalence rates of CP are observed in women, individuals from lower socioeconomic backgrounds, military veterans, and people from rural areas. CP is a challenging condition that extends beyond physical discomfort, impacting various aspects of an individual's life [1–3].

CP, typically defined as pain lasting more than three months, can be classified into nociceptive, neuropathic, and nociplastic pain, the latter resulting from a sensitized nervous system. CP is considered a central nervous system (CNS) disorder as it involves multiple neural networks, including sensory, emotional, cognitive, and behavioral elements [4-6]. The underlying pathophysiological mechanisms of CP include peripheral and central sensitization (the nervous system becomes hypersensitive to pain stimuli). Significant changes in neurotransmitter pathways and structural alterations in brain regions play a role in pain modulation. Two major neurotransmitter pathways, adrenergic and serotoninergic, are involved in CP pathophysiology. These intricate mechanisms contribute to the persistence and amplification of pain signals. Genetic variations in neurotransmitter pathways can influence pain sensitivity and the risk of developing CP. Moreover, CP is associated with morpho-functional changes in brain structures, mainly in the corticolimbic system. These changes are found in the amygdala, medial prefrontal cortex, periaqueductal gray, and anterior cingulate cortex, structures responsible for pain modulation and emotional responses [2,4,7,8].

The progression from acute to chronic pain involves neural plasticity, where persistent nociceptive signals result in long-term alterations in brain activity. This reorganization impacts pain processing, shifting it from sensory regions to emotional and limbic regions, leading to the chronicization of pain.

Because of these mechanisms, CP is closely linked to depression, anxiety, and mood disorders. These emotional challenges can aggravate the perception of pain, along with reduced physical function, limited mobility, and reduced daily activities, further affecting the overall quality of life and potentially leading to increased mortality rates [1,2,4,9].

Eye tracking is a reliable, non-invasive, and efficient method to assess brain functions related to oculomotor function, pupillometry, and blink analysis. It can evaluate multiple brain circuits involved in sensory, motor, and cognitive tasks. Abnormalities in eye movements, such as saccades, can serve as early indicators of neurological disorders. Eye tracking provides a direct measure of visual attention, often impaired in neurological conditions, and helps identify attentional biases and deficits, offering insights into the cognitive aspects of CP [10-13].

The study of blinks primarily focuses on spontaneous blink rate (SBR), a reliable biomarker of central dopaminergic activity. This is particularly relevant in CP research due to the role of dopamine in pain modulation. Analysing SBR can reveal insights into the dopaminergic mechanisms underlying CP by establishing how it influences pain perception and modulation. Integrating eye tracking and blink analysis, we can gain comprehensive insights into the neuronal networks of CP, aiding early diagnosis, monitoring, and treatment [14,15].

Our study aimed to offer a comprehensive framework for studying the interplay between blink rate, pain perception, and psychological factors in individuals experiencing CP.

## MATERIAL AND METHODS

This observational, retrospective study was conducted as part of a doctoral project at Iuliu Hațieganu University of Medicine and Pharmacy. This study was conducted in conformity with the principles of the Declaration of Helsinki. The dataset used in this study was provided by RoNeuro Institute for Neurological Research and Diagnostic. Given the retrospective nature of the study and the absence of any intervention on patients, there was no need for specific insurance for study participants. Confidentiality of information was ensured by not collecting any direct identifying data. Access to anonymized data was strictly limited to conducting retrospective analysis, ensuring that the subjects involved could not be identified or reidentified. Access to the data was limited only to the research team.

Ninety-two patients (24 men and 68 women) diagnosed with CP were included in the present analysis. Participants were assessed at the RoNeuro Institute for Neurological Research and Diagnostics in Cluj-Napoca, Romania, between February 2019 and March 2019. The evaluations included comprehensive clinical neurological, neuropsychological, and eye-tracking assessments.

Inclusion and exclusion criteria were rigorously applied to our database of neurological patients. Eligible individuals were required to have a confirmed diagnosis of CP and be between 18 and 80 years old at the time of the eye-tracking assessment. Exclusion criteria included a history of stroke or traumatic brain injury, severe psychiatric conditions, severe systemic disorders, terminal malignancies, or acute or advanced chronic ophthalmological diseases. Only patients who completed both the neurological evaluations and eye-tracking assessments were included in the analysis to ensure the reliability and accuracy of the data.

Neurological and psychological evaluations of these subjects were performed by board-certified professionals. Eye tracking evaluation consisted of a horizontal and a vertical visually guided saccadic task (VGST), each lasting approximately 5 minutes. Recordings were performed using the Tobii TX300 eye-tracking system (Tobii Technology, Stockholm, Sweden) [16]. Eye movement tasks were programmed using Tobii Studio 3.4.8 software and displayed on a 23-inch screen (16:6 aspect ratio, 1920x1080 pixel resolution, 60Hz refresh rate, 5ms response time). Tobii Tx300 device provided a 0.4° average gaze accuracy and a maximum processing latency of 3.3 ms [16,17].

Before each eye-tracking recording session, patients received task instructions, confirmed their understanding, and completed a series of practice trials. A 9-point calibration procedure was performed before each task to further ensure the quality of the recordings. The eye tracking system recorded binocular gaze data every 4 ms. Environmental conditions were also considered as patients were examined in a quiet, dimly lit room to limit external influences on gaze behavior. Subjects were positioned 65 cm away from the screen, and head movements were restricted using a chin and forehead rest. The visual stimulus displayed on the screen consisted of a red dot, 0.4° in diameter with a luminance of 63 cd/m^2^, against a black background with a luminance of 2.5 cd/m^2^ [18].

The trial structure followed a well-documented gap paradigm for eliciting visually guided saccades. Each trial started with a central fixation dot displayed for 1500ms, followed by a 200ms gap period (black background), and ended with the display of an eccentric dot displayed for 1500ms at different locations according to task specificity, at +/-10 and +/-18° visual angles in the VGST and 8° upward and downward along the central vertical axis in vertical VGST. The target location was randomized to avoid predictability. A total of 40 trials were used for each of the two tested paradigms, with an equal distribution of trials for each target amplitude. Participants were instructed to fixate their gaze on the central dot target throughout the recording.

The Tobii IV-T fixation filter was applied with specific parameters: average data from both eyes was used for eye selection, the velocity window length was set to 20 ms, and gap-fill interpolation was configured at 75 ms. Adjacent fixations were merged if the interval between them was 75 ms or less and the angular distance was 0.5° or less. The minimum fixation duration was established at 60 ms, and the I-VT classifier threshold was 30°/s, with noise reduction turned off. The exported recordings from Tobii Studio underwent further analysis via a custom-built platform that automatically determined saccadic parameters, enabled visual inspection of eye movements, and identified and counted valid blinks during the recording. Blinks were identified from the raw data as intervals where the validity of both left and right eye identification was 0 (high confidence) [17-20].

For this study, the primary eye-tracking parameter analyzed was the blink rate during horizontal and vertical (VGST). Blink rate is defined as the number of blinks a subject performs per minute [21].

To characterize the 92 CP patients included in our study, we collected data on age and sex. Pain perception was quantified using two validated instruments: the Central Sensitisation Inventory - Part A (CSI-A) and the McGill Pain Questionnaire (MPQ). The Central Sensitisation Inventory (CSI) is a self-reported questionnaire that identifies symptoms associated with central sensitization, and its psychometric properties, including construct validity, test-retest reliability, and internal consistency, make it a valuable tool for evaluating CP and developing tailored treatment strategies for patients with CS-related conditions [22–25]. The MPQ is a comprehensive tool that assesses the multidimensional aspects of pain intensity and quality through various descriptors and categories [26,27]. In addition, anxiety levels were measured using the State-Trait Anxiety Inventory (STAI), a widely used tool that assesses anxiety as both a current state and a longer-term trait in various settings, including mental health, research, and clinical evaluations [28].

Our database structure included the following variables: sex, age of the subject at evaluation, measured in years (age), blink rate during horizontal saccades (HS_BR), blink rate during vertical saccades (VS_BR), CSI_A – Central Sensitization Inventory – Part A (CSI_A), McGill Pain questionnaire score (McGill), State-Trait Anxiety Inventory – STATE (STAI-X1), State-Trait Anxiety Inventory – TRAIT (STAI-X2).

Statistical analyses were performed using R version 4.3.2 and RStudio, using key libraries such as 'openxlsx' - spreadsheet file interaction, 'ggplot' and 'patchwork' for creating graphical representations. All analyses were performed with a significance threshold set at alpha = 0.05. Pearson and Spearman correlation analyses were carried out to explore the relationships between variables.

## RESULTS

Our analysis included blink rate evaluation during VGST for 92 patients with CP. We included 24 men (26.09%) and 68 women (73.91%). The age distribution of participants can be observed in Table 1. Descriptive statistics for blink rate measured during horizontal and vertical VGST are presented in Table 2.

**Table 1:** Age distribution of patients with chronic pain

	Age at eye tracking evaluation		
	All	Men	Women
Mean	40	36	42
SD	13.69	12.17	13.94
Min	18	18	18
Max	68	59	68
Range	50	41	50

**Table 2:** Blink rate distribution

Blink Rate	Mean	SD	Min	Max
HS_BR	12,40	9.48	1	49.14
VS_BR	11.83	9.06	1.56	58.52

**Figure 1:**
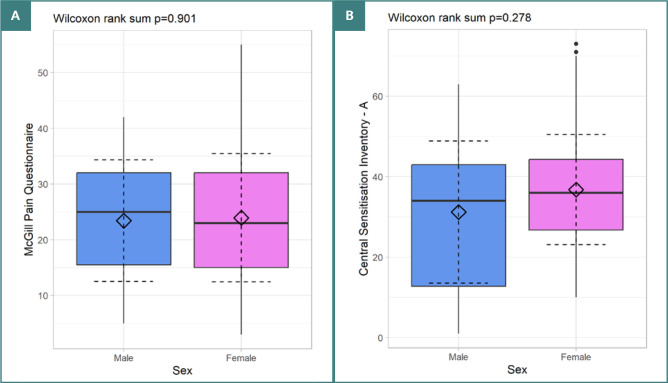
Distribution of pain perception scores by sex. A, MPQ scores. B, CSI-A scores.

The CSI-A had a mean score of 35.32 (SD = 14.91), while the MPQ had a mean score of 23.80 (SD = 11.32). We analyzed the distribution of pain perception scores between males and females using these scales and found no significant differences between the sexes in our sample of patients with CP, as illustrated in Figure 1.

**Figure 2:**
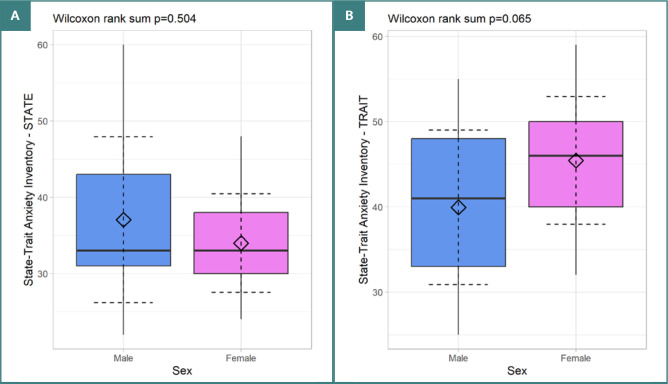
Distribution of STAI scores by sex A. Distribution of STAI-STATE scores. B, Distribution of STAI-TRAIT scores.

Out of the 92 patients, 42 successfully completed the STAI test. Consequently, the analysis and descriptive statistics for the STAI test are based on this smaller subgroup. The STAI-State scores had a mean of 35.23 (SD = 8.54), while the STAI-Trait scores had a mean of 43.21 (SD = 8.50). The distribution of these scores by sex is illustrated in Figure 2.

**Figure 3:**
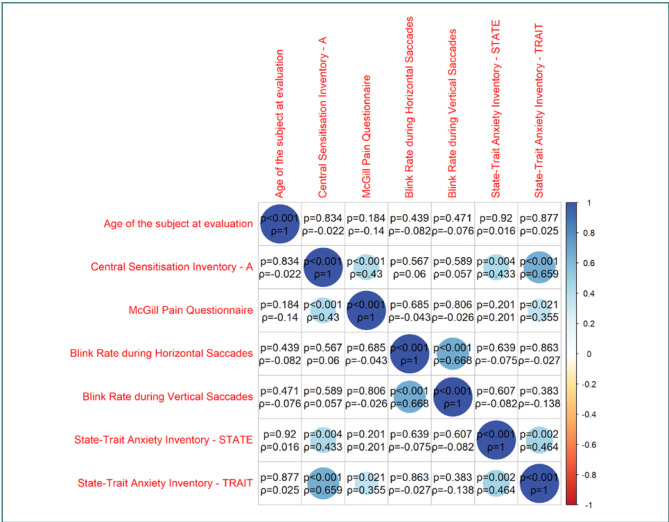
Correlation matrix of the studied variables

Building on our descriptive statistics findings, we conducted a detailed analysis to explore potential correlations among the studied variables. The results of this correlation analysis are presented in the correlation matrix (Figure 3).

**Figure 4:**
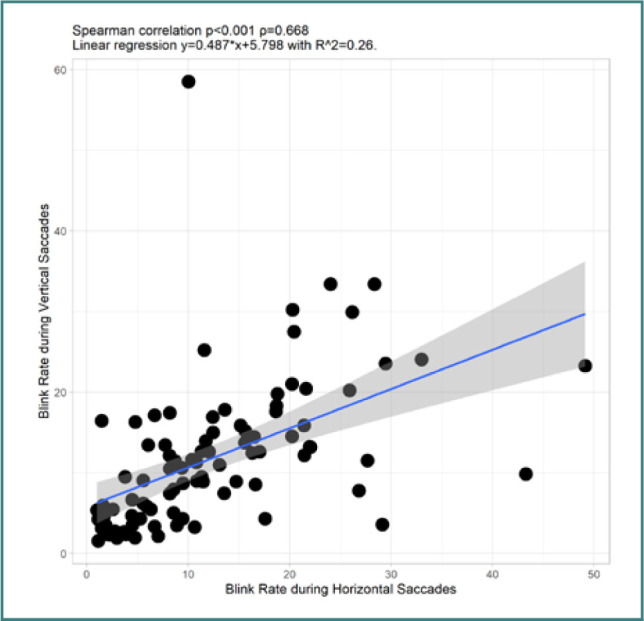
Correlation between blink rate during horizontal VGST and vertical VGST

Our analysis showed no correlation between age and other variables. However, blink rate during horizontal VGST showed only one statistically significant result, a highly positive correlation with blink rate during vertical VGST (*P* <0.001, ρ = 0.668) – Figure 4. The blink rate did not correlate with age, CSI_A, MPQ, or STAI scores.

**Figure 5:**
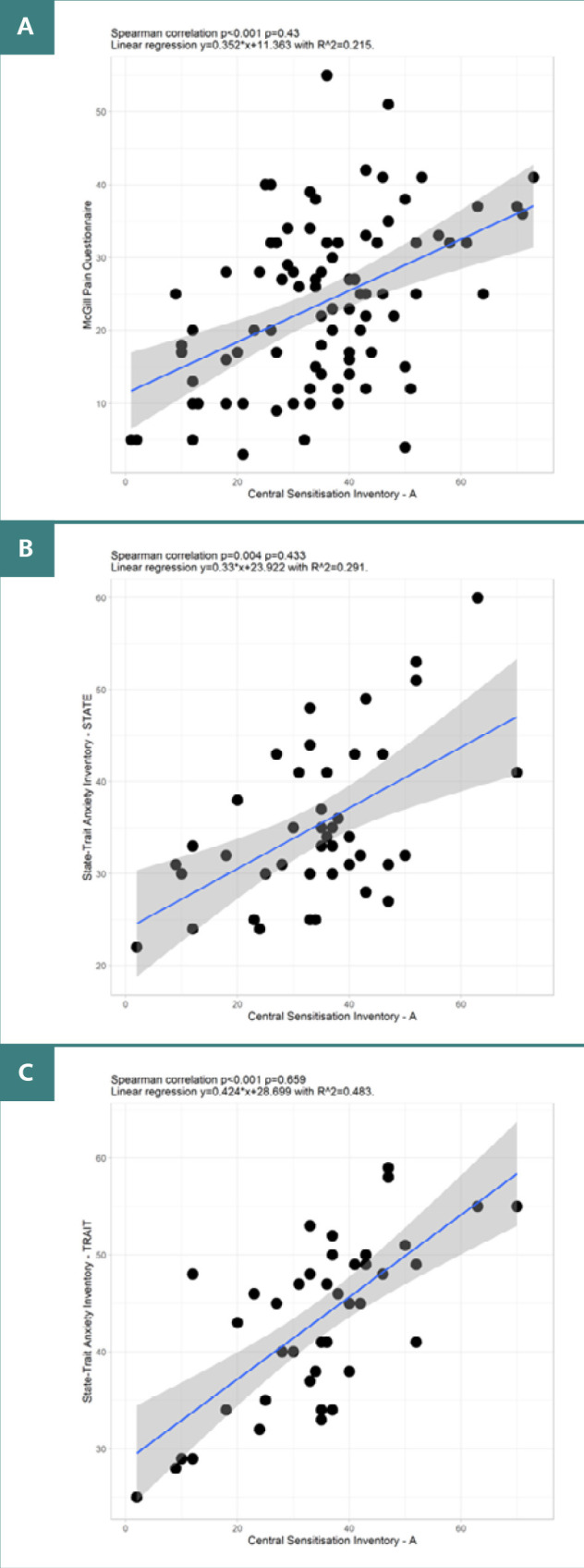
Correlations between CSI-A and pain/anxiety measures. A, Correlation between CSI-A and MPQ. B, Correlation between CSI-A and STAI–STATE. C, Correlation between CSI-A and STATE–TRAIT

The two scales used to quantify pain perception showed the following correlation: the CSI – PART A showed a moderate positive correlation with the MPQ (*P* < 0.001, ρ = 0.43) and STAI STATE (*P* = 0.004, ρ =0.433) and a high positive correlation with STAI TRAIT (*P* = < 0.001, ρ = 0.659) – see Figure 5. Lastly, STATE_X1 showed a moderate positive correlation with STATE_X2 (*P* = 0.002, ρ =0.464)

## DISCUSSION

Our comprehensive analysis of 92 patients with CP identified several significant correlations among the studied variables. For example, there was a strong positive correlation between blink rates during horizontal and vertical VGST. Additionally, the central sensitization scores demonstrated significant positive correlations with both the MPQ and the two STAI scores. Furthermore, we observed a positive correlation between the two anxiety tests applied.

Blinks are often linked to cognitive and neurological processes controlling eye movements [29,30]. The coordination of blinks with saccadic movements is well-documented. Both horizontal and vertical visually guided saccades are governed by similar neural mechanisms, explaining the correlation in blink rates [21,31–33]. We observed a lack of correlation between pain perception and blink rate during saccades, suggesting that central sensitization processes do not directly influence blink rate during saccadic tasks. While CP can influence many physiological and psychological factors, the neural circuits controlling saccadic eye movements and blinking might not be directly affected by the subjective, self-reporting aspects of pain.

A higher CSI score is an indicator that the central nervous system becomes hyper-reactive, leading to an amplification of pain signals [34,35]. Patients with central sensitization often experience heightened pain sensitivity, widespread pain, and pain from stimuli that are generally not painful (allodynia) [36,37]. The MPQ measures various dimensions of pain, including sensory and affective aspects. In patients with central sensitization, these dimensions of pain are often exaggerated due to the hyper-reactive state of the nervous system [38,39]. The CSI and the MPQ assess aspects of the same underlying pathophysiological mechanisms. The CSI is specifically designed to identify symptoms related to central sensitization, while the MPQ assesses the overall pain experience, which can be heavily influenced by central sensitization. Therefore, higher CSI scores are likely associated with higher MPQ scores.

Heightened sensitivity, as indicated by high CSI scores, can amplify pain perception and contribute to emotional distress, including anxiety [38,40,41]. Both anxiety and central sensitization involve dysregulation of the same neurobiological pathways, including alterations in neurotransmitter systems and increased activity in brain regions associated with pain and emotion, such as the amygdala and prefrontal cortex [4,42]. Living with CP is a significant source of ongoing stress, which can exacerbate feelings of anxiety. The persistent nature of CP can lead to a state of hypervigilance and anxiety about pain flare-ups and their impact on daily life. Patients with CP may develop maladaptive thought patterns and emotional responses, such as catastrophizing, fear of pain, and helplessness, all of which are closely linked to higher anxiety levels. CP often limits physical activity and social participation, contributing to higher anxiety. Pain can interfere with sleep quality, leading to sleep deprivation, which has been shown to increase anxiety levels [1–3]. Both the CSI and STAI scales rely on self-reported data, which means that the subjective experience of distress can influence scores on both measures.

While state anxiety measures the current level of anxiety in a specific situation, and trait anxiety measures a person's general tendency to experience anxiety, there is naturally some overlap between these constructs [28,43]. People who generally have higher levels of anxiety (trait anxiety) are more likely to experience higher levels of anxiety in specific situations (state anxiety). The moderate positive correlation between state and trait anxiety scores in patients with CP underscores the intertwined nature of temporary and long-lasting anxiety responses in the context of CP.

Our study is faced with several limitations. First, the retrospective, observational design of the study naturally restricts our ability to draw conclusions about causality. Secondly, the relatively small sample size (*n* = 92) may not provide sufficient statistical power to detect smaller effects or generalize the findings to a larger population. In addition, we did not perform subgroup analysis based on neurological diagnosis. Moreover, our analysis was restricted to blink rate without taking into consideration other blink analysis variables that could offer more comprehensive insights into blinking particularities. We only examined blinks during visually guided saccadic tasks without correlating with saccadic eye movement. Future research should focus on longitudinal studies with larger cohorts to validate and extend our findings. Incorporating neuroimaging techniques and EEG could provide a deeper understanding of the underlying structural and functional neural mechanisms while applying a wider array of eye-tracking paradigms, including memory-guided saccades and visual search performance tasks that could offer more insights on blink rate.

## CONCLUSION

Our analysis offers a broad view of blink rate, subjective measures of pain intensity, and anxiety. Understanding these correlations lays the foundations for more tailored and effective interventions for managing chronic pain and its invalidating impact on the quality of life.
